# Improving Diagnostic Sensitivity in Imbalanced Oral Cancer Image Classification: A Comparative Study of CNN and Transformer Architectures

**DOI:** 10.3390/cancers18182926

**Published:** 2026-09-09

**Authors:** Pablo Ormeño-Arriagada, Valentina Zúñiga, Carlos Toro, Gastón Márquez, David Araya, Diego Mellado, Carla Taramasco

**Affiliations:** 1 Instituto de Tecnología para la Innovación en Salud y Bienestar, Facultad de Ingenieria, Universidad Andres Bello, Viña del Mar 2530958, Chilediego.mellado@unab.cl (D.M.);; 2 CECAN, Centro para la Prevención y Control del Cancer, Santiago 8330077, Chile; 3 Faculty of Engineering, Universidad Andres Bello, Autopista Concepción-Talcahuano CCP-THNO 7100, Talcahuano 4260000, Chile; carlos.toro@unab.cl; 4 Departamento de Ciencias de la Computación y Tecnología de la Información, Universidad del Biobio, Chillán 4081112, Chile

**Keywords:** oral cancer, class imbalance, data augmentation, convolutional neural networks, vision transformer, malignant-risk detection, medical image classification

## Abstract

Background: Class imbalance remains a major limitation in artificial intelligence based oral cancer diagnosis, particularly in small clinical image datasets where malignant lesions are underrepresented. Methods: Using a dataset of 3000 mobile-acquired oral cavity images categorized into four diagnostic classes (healthy, benign, oral potentially malignant disorders, and oral cancer), we systematically evaluated the impact of imbalance mitigation strategies on diagnostic performance. Three backbone architectures, EfficientNet, Vision Transformer, and Swin Transformer, were trained under four conditions: raw imbalanced training, random under-sampling, random over-sampling, and medical-safe data augmentation. Performance was evaluated using stratified five-fold cross-validation, and all reported metrics correspond to the mean performance across the validation folds. Results: In multiclass evaluation, augmentation yielded the strongest overall performance, with EfficientNet achieving the highest macro-$F_{1}$ score (0.669 ± 0.024) and area under curve (0.881 ± 0.015). Under clinically oriented binary malignant-risk evaluation (high-risk vs. low-risk lesions), sensitivity reached 0.817 ± 0.030 with EfficientNet, while data augmentation maintained competitive malignant-risk detection across the evaluated architectures. In contrast, random undersampling consistently yielded lower descriptive performance across architectures. Conclusions: These findings indicate that augmentation-based imbalance mitigation is associated with improved multiclass and oral cancer specific performance while maintaining competitive overall discrimination. The results provide practical guidance for developing clinically robust artificial intelligence systems for early oral cancer detection in imbalanced real-world datasets.

## Simple Summary

Early detection of oral cancer is essential for improving patient survival, yet artificial intelligence models often struggle because available clinical image datasets are small and highly imbalanced, with relatively few malignant cases. This study investigates whether class imbalance can be mitigated through random under-sampling, random over-sampling, and medically informed image augmentation when training three representative deep learning architectures: EfficientNet, Vision Transformer, and Swin Transformer. Using a publicly available dataset of oral cavity photographs and a unified five-fold cross-validation framework, we compared these strategies using both multiclass classification and a clinically oriented malignant-risk evaluation. The results indicate that data augmentation generally improves minority-class detection while maintaining competitive overall classification performance. Nevertheless, external multicenter validation and prospective clinical studies are required before routine clinical application.

## 1. Introduction

### 1.1. Background and Clinical Motivation

Oral cancer (OCA) represents a significant global health burden, ranking among the most common cancers in low and middle income countries [1]. Early detection is critical because the 5-year survival rate drops drastically, from over 80% in early stages to less than 30% in advanced stages [2]. Despite being accessible to visual and tactile examination, oral cancers are frequently diagnosed late due to subtle early-stage symptoms and limited screening programs [3]. Early identification not only improves survival but also reduces treatment-related morbidity and healthcare costs [4]. Integrating artificial intelligence (AI) diagnostic tools into clinical workflows has the potential to assist clinicians in identifying malignant lesions earlier and more accurately, thereby enhancing prognostic outcomes and guiding timely interventions [5].

### 1.2. Problem Statement

Oral cancer image classification remains challenging due to limited curated datasets and intrinsic class imbalance across diagnostic categories [6]. In clinical practice, malignant and early-stage lesions are frequently underrepresented, creating skewed distributions that bias deep learning (DL) models toward majority classes. Consequently, the combination of imbalance and small sample sizes restricts generalization, increases overfitting risk, and may yield high overall accuracy while concealing poor sensitivity for clinically significant lesions [7]. This limitation is particularly important because false negatives can delay diagnosis and adversely affect patient outcomes [8,9]. Therefore, imbalance-aware strategies have become essential for developing clinically relevant diagnostic systems. Approaches such as random oversampling (ROS) and medically informed image augmentation aim to improve minority representation and strengthen model robustness under realistic acquisition conditions [10,11,12,13,14,15]. Collectively, these methods offer a practical pathway toward more equitable oral cancer classification.

### 1.3. Research Gap

Although numerous studies have applied DL to oral cancer image classification, most focus on architectural refinement and feature extraction while giving limited attention to class imbalance. Consequently, reported performance frequently relies on overall accuracy, a metric that may obscure reduced sensitivity for minority lesion categories, including early-stage malignancies. Moreover, conventional mitigation strategies often rely on loss reweighting or simple oversampling, providing only partial correction. Advanced use of oversampling and medically appropriate data augmentation remain insufficiently explored [16]. To address this gap, the present study systematically evaluates oversampling and augmentation strategies across modern backbone architectures in small and imbalanced oral cancer datasets [13,15,17]. Accordingly, this study systematically evaluates the contribution of oversampling and medically informed data augmentation across representative convolutional and transformer-based architectures under a unified experimental framework.

### 1.4. Study Aim

The aim of this study is to evaluate whether oversampling and data augmentation can mitigate the limitations imposed by small and imbalanced oral cancer image datasets. Specifically, we investigate the effectiveness of ROS and medically informed data augmentation across three representative backbone architectures, Vision Transformer (ViT), Swin Transformer, and EfficientNet. This framework enables a controlled assessment of imbalance-aware training for the detection of rare malignant lesions under clinically realistic conditions. Furthermore, we examine whether the integration of sampling and augmentation strategies improves minority-class sensitivity while preserving overall discrimination performance. Through ablation experiments and statistical comparison, we seek to provide evidence-based guidance for the development of clinically meaningful diagnostic models. Ultimately, this work aims to support safer and more equitable future clinical evaluation of AI-driven oral cancer screening systems.

### 1.5. Hypotheses and Research Questions

To guide the experimental evaluation, the following hypothesis and research questions were formulated:

In small and imbalanced oral cancer image datasets, augmentation-based imbalance mitigation yields improved multiclass and malignant-risk classification performance compared with conventional resampling strategies (RUS and ROS), resulting in improved sensitivity, macro-$F_{1}$ score, and classification stability across convolutional and transformer-based architectures.

The research questions can be formulated as follows:

- RQ1: How does data augmentation compare with random under-sampling (RUS) and random over-sampling (ROS) in improving multiclass diagnostic performance under class imbalance?
- RQ2: Does augmentation-based training improve malignant-risk detection (OCA + oral potentially malignant disorders (OPMD)) under a clinically oriented binary evaluation setting?
- RQ3: Are convolutional architectures more robust than transformer-based architectures when trained on small and imbalanced oral cancer image datasets?
- RQ4: How does data augmentation influence class-specific recall and confusion patterns among oral lesion categories under multiclass classification?

### 1.6. Contributions

The main contributions of this work are fourfold. First, we present a unified framework that integrates class imbalance mitigation with deep learning for oral cancer image classification under limited and imbalanced data conditions, enabling systematic comparison of EfficientNet, Vision Transformer, and Swin Transformer. Second, we evaluate Random Oversampling and medically informed data augmentation through controlled experiments to quantify their impact on minority-class detection and overall diagnostic performance. Third, we perform a clinically oriented evaluation by combining multiclass classification with malignant-risk screening using sensitivity, specificity, balanced accuracy, macro-$F_{1}$ score, and Cohen’s kappa. Finally, we promote performance consistency across cross-validation folds through comprehensive reporting of preprocessing, augmentation, validation protocols, and implementation details. Unlike previous studies emphasizing architectural improvements or overall accuracy, this work systematically evaluates imbalance mitigation strategies and their effect on clinically relevant diagnostic performance. Distinct from previous studies that primarily compare model architectures or optimize overall classification accuracy, the present work focuses specifically on class-imbalance mitigation under a unified experimental protocol. Its unique contribution is the controlled comparison of RUS, ROS, and medically informed data augmentation across convolutional and transformer-based architectures, complemented by multiclass, malignant-risk, OCA-specific, and fold-wise statistical analyses. This design enables the effects of imbalance mitigation to be examined independently of changes in model architecture or evaluation protocol.

### 1.7. Organization of the Paper

The remainder of this paper is organized as follows. Section 2 reviews prior work on oral cancer image classification and imbalance mitigation strategies. Section 3 describes the dataset, backbone architectures, imbalance handling techniques, and evaluation protocol. Section 4 presents multiclass and binary clinical performance results. Section 5 interprets the findings in relation to clinical applicability and methodological considerations. Finally, Section 6 summarizes the principal contributions and outlines future research directions.

## 2. Related Work

Recent advances in artificial intelligence have accelerated the development of automated oral cancer image analysis systems. While substantial progress has been achieved through convolutional and transformer-based architectures, comparatively limited attention has been given to imbalance mitigation and its implications for clinically meaningful diagnostic performance.

### 2.1. Artificial Intelligence in Oral Cancer Image Analysis

Recent advances in AI have enabled automated analysis of oral lesion images for early detection of oral squamous cell carcinoma (OSCC) [18]. Convolutional neural networks (CNNs) have demonstrated promising performance in classifying benign and malignant oral lesions from photographic datasets. Studies employing architectures such as ResNet, EfficientNet, and DenseNet have reported competitive accuracy and area under the ROC curve (AUC) metrics in controlled datasets. More recently, transformer-based architectures, including ViT and Swin Transformer [19], have been explored in medical imaging tasks due to their capacity to model long-range dependencies. However, their application in oral cancer classification remains comparatively limited, particularly under constrained data conditions [20].

Recent systematic reviews and meta-analyses have reported high pooled diagnostic accuracy for AI-based oral cancer detection while identifying substantial heterogeneity across datasets, class distributions, and validation protocols [21]. These findings suggest that performance variability is strongly influenced by class imbalance and limited external validation, underscoring the need for standardized evaluation frameworks and screening-oriented sensitivity analyses. Although both clinical photography and histopathological imaging have been extensively investigated, they address distinct diagnostic tasks. Histopathological images characterize microscopic tissue morphology after biopsy, whereas clinical photographs depict macroscopic oral lesions acquired under real-world conditions. Accordingly, this review focuses on studies using clinical oral photographs, while histopathological investigations are considered complementary methodological references rather than direct performance benchmarks [20].

### 2.2. Challenges of Class Imbalance in Medical Imaging

Imbalanced training data can inflate overall accuracy while masking poor minority-class detection. In cancer diagnosis, such bias may compromise deployment because missed malignant cases carry significant clinical consequences [22,23]. A further concern relates to the distortion of diagnosis decision thresholds under imbalanced conditions. When malignant cases are scarce, models may adopt conservative decision boundaries that favor specificity at the expense of sensitivity, undermining early detection efforts. Such imbalance-driven threshold shifts can compromise risk stratification and triage performance in screening contexts. Recent analyses highlight that imbalance not only affects predictive metrics but also influences clinical utility and deployment safety in oncologic AI systems [24].

### 2.3. Resampling Strategies for Imbalance Mitigation

To address class imbalance, conventional resampling techniques include ROS, which duplicates minority samples, and RUS, which removes majority samples. Although simple to implement, ROS may increase overfitting, whereas RUS can result in information loss [25]. Synthetic approaches such as SMOTE have also been widely applied to tabular medical data, although their suitability for image-based deep learning remains context-dependent [26]. More recently, hybrid and feature-space strategies have incorporated minority enhancement within embedding representations or combined resampling with augmentation and cost-sensitive learning. These approaches aim to improve minority discrimination while preserving anatomical realism. However, their effectiveness remains dependent on dataset size and heterogeneity, highlighting the need for systematic evaluation under realistic medical imaging conditions [27].

### 2.4. Data Augmentation in Small Medical Image Datasets

Image data augmentation is widely used to increase effective dataset size and improve deep learning generalization. In medical imaging, however, augmentation must preserve pathological characteristics while introducing realistic variability through controlled transformations such as rotation, brightness adjustment, and scaling [28]. Although augmentation has demonstrated benefits across cancer imaging applications, its comparative effectiveness against conventional resampling strategies in oral cancer classification remains insufficiently evaluated [29]. Excessive or poorly controlled transformations may distort diagnostically relevant color, texture, lesion boundaries, or morphology, thereby compromising clinical realism. Consequently, augmentation protocols should be medically informed and context-aware, particularly in small oncologic datasets where subtle visual features may be diagnostically important [30]. These considerations support the use of carefully constrained augmentation rather than generic image perturbations.

### 2.5. Research Gap and Contribution

Despite growing interest in AI-based oral cancer detection, few studies have systematically compared imbalance mitigation strategies across convolutional and transformer architectures. Patient-level data leakage also remains a concern because correlated images from the same participant may appear across training and validation partitions, while public datasets often lack identifiers required for patient-wise partitioning. Accordingly, this study compares RUS, ROS, and medically informed data augmentation using EfficientNet, ViT, and Swin Transformer. Unlike studies emphasizing multiclass accuracy, clinically oriented malignant-risk evaluation remains limited despite its relevance to screening [31]. The proposed framework therefore integrates multiclass and binary malignant-risk analyses under a unified protocol. While recent studies demonstrate effective deep learning-based oral cancer classification [32,33], methodological differences limit direct numerical comparison. Table 1 summarizes representative studies and contrasts their methodological characteristics with the proposed framework.

## 3. Materials and Methods

This section describes the dataset, model architectures, imbalance mitigation strategies, and evaluation procedures employed in this study. A unified experimental framework was designed to systematically compare convolutional and transformer-based architectures under controlled cross-validation, ensuring fair assessment across imbalance handling strategies.

### 3.1. Study Design and System Architecture

**Figure 1:**
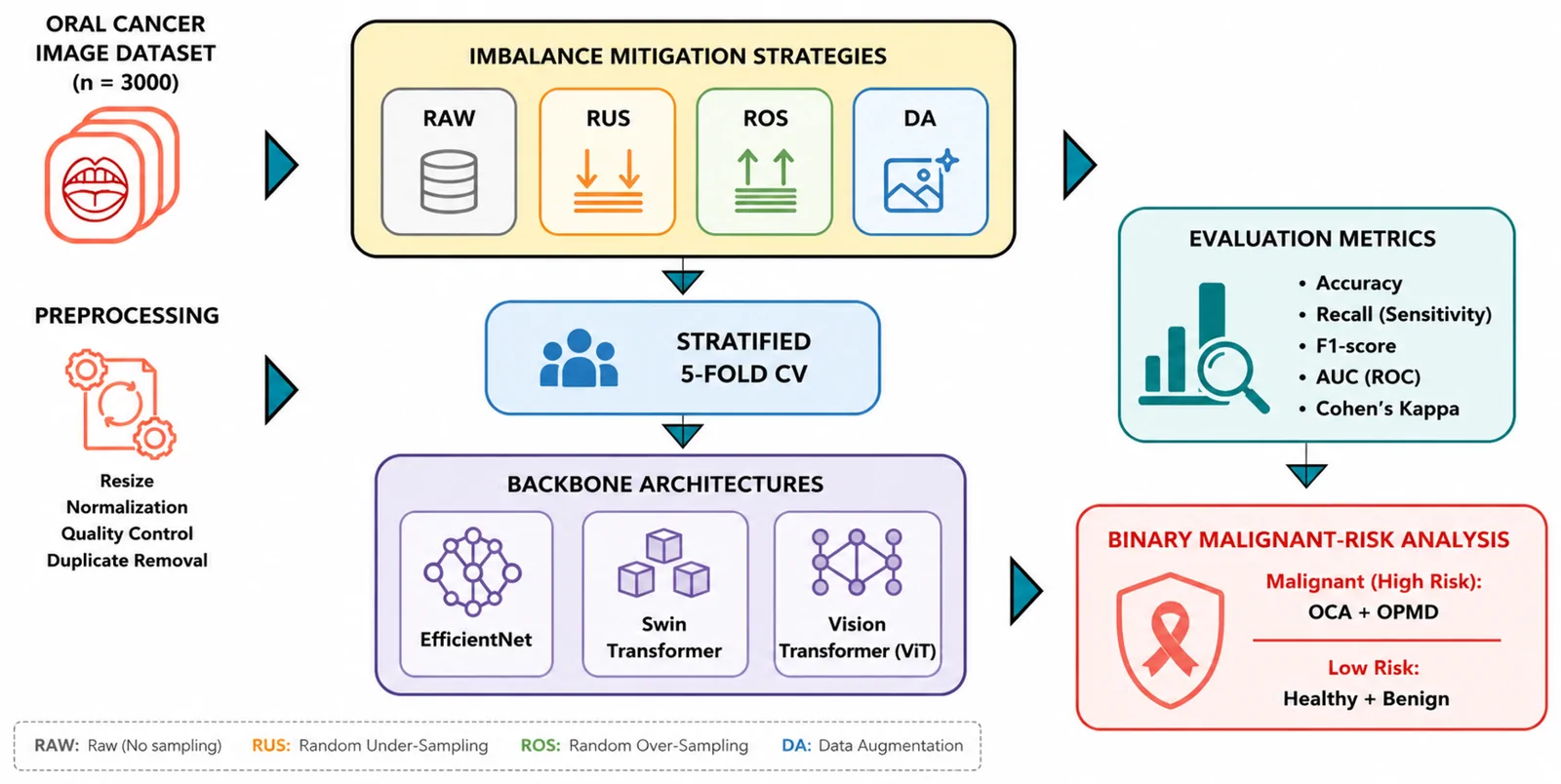
Overview of the study workflow. Oral cavity images were preprocessed and evaluated using stratified five-fold cross-validation under four imbalance mitigation strategies (RAW, RUS, ROS, and DA). EfficientNet, Swin Transformer, and Vision Transformer (ViT) were compared using multiclass classification metrics and a binary malignant-risk evaluation grouping OCA and OPMD versus healthy and benign lesions. This figure is a conceptual workflow diagram and does not represent generated or simulated experimental results.

Figure 1 illustrates the current experimental workflow adopted in this study. Oral cavity images were first preprocessed and then evaluated using a stratified five-fold cross-validation framework under four class imbalance conditions: the original dataset, RUS, ROS, and DA. Each strategy was independently applied to EfficientNet, Swin Transformer, and ViT, and model performance was assessed using both multiclass classification metrics and a clinically oriented binary malignant-risk evaluation. The pipeline enforces strict separation between training and validation folds to minimize information leakage, with imbalance mitigation applied exclusively to the training data. Performance was subsequently evaluated using standardized metrics to quantify classification accuracy, agreement, and clinically relevant screening trade-offs. Because unique patient identifiers were unavailable in the dataset, cross-validation was necessarily performed at the image level rather than the patient level. Consequently, patient-level independence across folds could not be verified. To minimize procedural data leakage, all resampling and augmentation operations were performed exclusively within the training partition of each fold, whereas validation images remained unchanged and were not used during imbalance mitigation.

### 3.2. Dataset Description

The dataset comprises 3000 high-resolution oral cavity images acquired using mobile phone cameras from the Sri Lankan population. Expert clinicians categorized each image into one of four classes: Healthy, Benign, OPMD and OCA, with the class distribution summarized in Table 2. According to the original dataset documentation, OCA cases were confirmed by histopathological examination following biopsy, whereas OPMD, benign lesions, and healthy mucosa were assigned using the clinical diagnostic protocol established by the dataset authors. The present study adopted these reference labels without modification. In the original dataset, the OCA category corresponds exclusively to oral squamous cell carcinoma (OSCC); therefore, the terms OCA and OSCC are used interchangeably throughout this manuscript.

The dataset also includes a structured metadata file for each sample, providing clinically relevant patient information such as shown in Table 3:

Table 4 summarizes the demographic characteristics and prevalence of major risk factors across the four diagnostic categories, including sample size, mean age, sex distribution, smoking, betel quid chewing, and alcohol consumption. These variables provide epidemiological context for interpreting model performance. A Welch independent-samples *t*-test showed that individuals represented by OCA images were significantly older than those represented by images from the remaining diagnostic categories (t=12.80, df=151.16, p<0.0001, mean difference = 15.06 years, 95% CI = [12.74, 17.39]). Together, these findings characterize cohort composition and establish the clinical context for the subsequent evaluation of deep learning models.

This study utilized a publicly available, restricted-access dataset [37] originally collected under institutional ethics oversight by the University of Peradeniya, Sri Lanka, and published in [38]. Ethics approval for the original data collection and informed consent from all participants were obtained by the dataset authors; no new human data were collected in the present study. The dataset was accessed and used solely for non-commercial research purposes in accordance with its Creative Commons Attribution Non-Commercial No Derivatives 4.0 (CC BY-NC-ND 4.0) license. The publicly available dataset does not include unique patient identifiers. Consequently, it was not possible to determine whether multiple images originated from the same individual or to verify patient-level duplication. No independent image-level duplicate or near-duplicate screening beyond the dataset records was performed; therefore, the possibility of visually duplicated or highly similar images cannot be completely excluded.

### 3.3. Backbone Architectures

Three representative backbone architectures, Vision Transformer, Swin Transformer, and EfficientNet, were selected to compare attention-based and convolutional learning approaches under small and imbalanced oral cancer image datasets. ViT facilitates analysis of global feature modeling without convolutional inductive bias, offering insight into long-range contextual learning. The Swin Transformer introduces hierarchical representation with shifted window attention, integrating local aggregation and broader contextual awareness. EfficientNet serves as a robust convolutional baseline, employing compound scaling to balance parameter efficiency and stability under limited data conditions. Collectively, these architectures enable structured comparison of representational strategies within imbalance-aware training. Table 5 outlines their architectural characteristics and roles within the proposed classification framework. All backbone architectures were initialized using publicly available ImageNet-pretrained weights. Vision Transformer employed the Hugging Face implementation google/vit-base-patch16-224, Swin Transformer used microsoft/swin-base-patch4-window7-224, and EfficientNet-B0 was initialized with the ImageNet-pretrained weights provided by the PyTorch implementation.

### 3.4. Resampling Strategies

To address the class imbalance present in oral cancer image datasets, we employ two commonly used resampling strategies: Random Oversampling (ROS) and Random Undersampling (RUS). These methods increase the representation of minority classes, mitigating bias during training and improving classifier sensitivity. Table 6 summarizes their principles, advantages, and limitations.

### 3.5. Image Data Augmentation

Data augmentation mitigates overfitting and class imbalance in small medical image datasets by synthetically expanding the training distribution. During training, predefined geometric and photometric transformations were applied online using fixed probabilities and parameter ranges to increase image diversity while preserving diagnostically relevant anatomical characteristics. To address class imbalance, augmentation was applied exclusively to minority-class images within each training fold, leaving validation data and majority-class samples unchanged. Geometric transformations, including flipping, rotation, cropping, and resizing, simulated variations in viewpoint and camera positioning, whereas photometric transformations, such as color jittering, brightness modulation, blurring, and noise injection, reproduced realistic acquisition and illumination variability [15]. All transformations were constrained to preserve pathological realism, thereby increasing intra-class variability without altering diagnostic identity. Table 7 summarizes the augmentation categories, advantages, and limitations.

**Figure 2:**
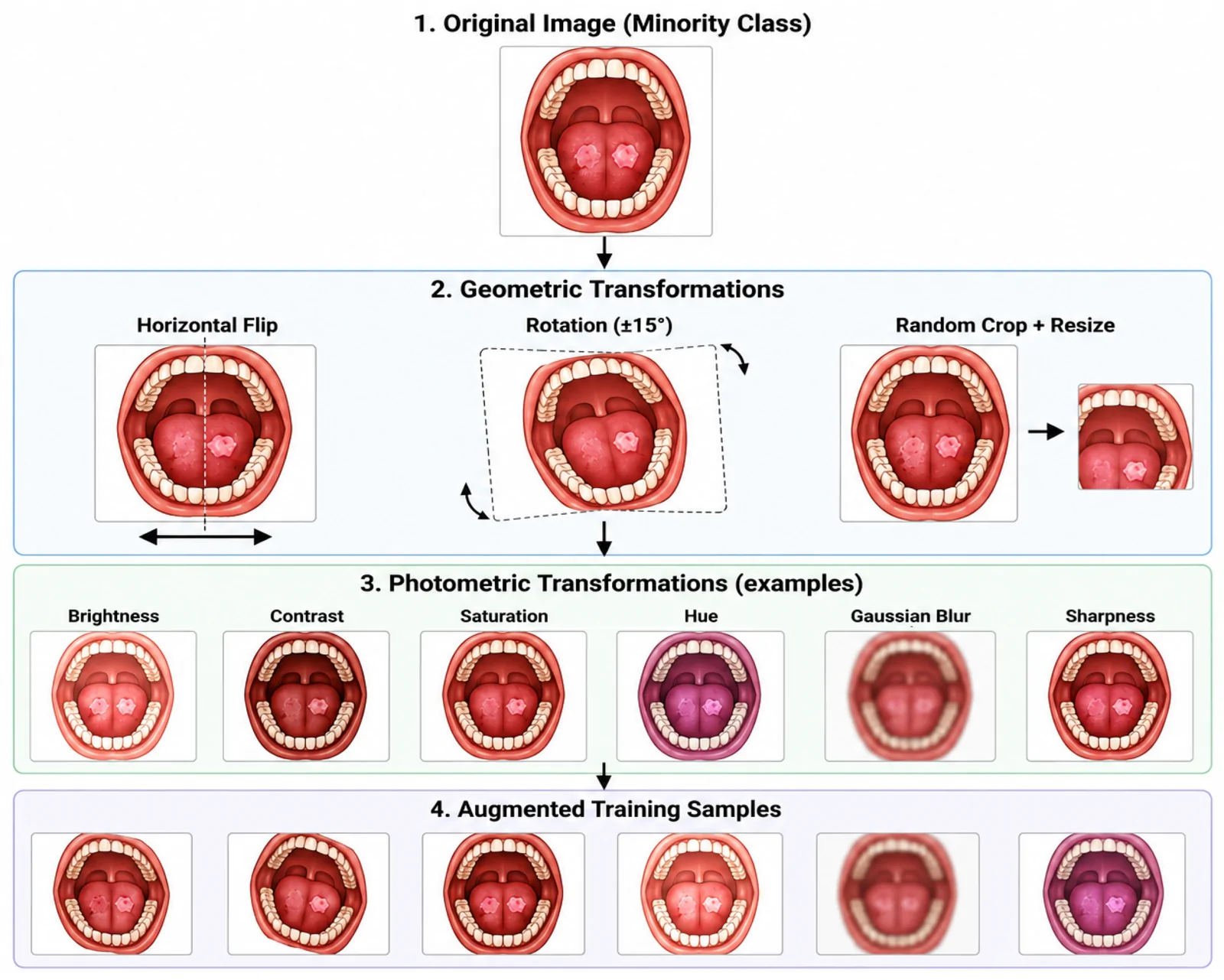
Conceptual schematic representation of the online data augmentation pipeline. Geometric transformations (horizontal flip, rotation, and random resized crop) and photometric transformations (brightness, contrast, saturation, hue, Gaussian blur, and sharpness adjustment) were applied exclusively to minority-class training images during stratified five-fold cross-validation. These operations increased training diversity while preserving clinically relevant lesion morphology. Validation images were not augmented. This schematic is illustrative only and does not contain or represent experimental measurements.

Figure 2 summarizes the augmentation workflow applied during training. The schematic illustrates how minority-class images were subjected to geometric and photometric transformations to generate diverse training samples while preserving clinically relevant lesion morphology. All augmentations were performed exclusively within the training partitions of each cross-validation fold, whereas validation images remained unchanged.

The data augmentation pipeline was designed to increase training diversity while preserving clinically relevant lesion morphology and color characteristics. Geometric transformations simulated variations in camera positioning and lesion orientation, whereas photometric transformations reproduced realistic differences in illumination, color, and image sharpness commonly encountered during mobile image acquisition. To avoid excessive image distortion, all augmentation parameters were constrained within conservative ranges based on commonly adopted practices in medical image analysis. Table 8 summarizes the transformations, parameter settings, and application probabilities used during model training.

All images were resized to match the input resolution required by each backbone architecture. Vision Transformer and Swin Transformer used an input resolution of 224×224 pixels through their default Hugging Face image processors, whereas EfficientNet-B0 employed image resizing followed by center cropping to 480×480 pixels. Pixel intensities were normalized using the standard ImageNet mean ([0.485, 0.456, 0.406]) and standard deviation ([0.229, 0.224, 0.225]). No manual lesion segmentation, lesion-centering procedure, background removal, or region-of-interest cropping was performed. Images were processed in their original clinical context before augmentation and model training.

### 3.6. Evaluation

A stratified 5-fold cross-validation strategy was employed to provide robust model evaluation while preserving the original class distribution. The dataset of 3000 images was partitioned into five approximately equal folds of 600 images each. During each iteration, one fold served as the validation set, whereas the remaining four folds (approximately 2400 images) were used for training. Final performance metrics were computed as the mean across all five folds. Because patient identifiers were unavailable, partitioning was performed exclusively at the image level. Model performance was evaluated using a comprehensive set of metrics for imbalanced multiclass classification, as summarized in Table 9. Together, these metrics quantify overall classification performance, minority-class detection, and chance-corrected agreement, enabling a consistent comparison of the evaluated imbalance mitigation strategies.

Calibration analysis was not performed in the present study, as model evaluation focused on threshold-based classification outputs derived from argmax decisions. While AUC was computed from softmax probabilities, reliability curves and expected calibration error (ECE) were not assessed. Future work should investigate probability calibration to ensure that confidence estimates align with true outcome frequencies, particularly for deployment in clinical decision-support settings. This is clinically important because poorly calibrated confidence estimates may lead to inappropriate risk thresholds, potentially increasing missed high-risk lesions or unnecessary referrals even when discrimination metrics such as AUC remain favorable.

### 3.7. Experimental Procedures

The experimental framework evaluated three backbone architectures, EfficientNet, Swin Transformer, and Vision Transformer, under four imbalance mitigation strategies: RAW, Random Undersampling (RUS), Random Oversampling (ROS), and Data Augmentation (DA). Images and labels were defined in *ImagewiseData.csv*. Models were trained using identical procedures under a stratified five-fold cross-validation protocol, with the same data partitions used across all architectures and experimental conditions to ensure fair comparison. The same stratified fold assignments were maintained across the evaluated architectures and imbalance mitigation strategies to ensure comparable evaluation conditions. No information from the validation partition was used to determine the training-set resampling or augmentation operations. Cross-validation was performed at the image level because patient identifiers were unavailable. Resampling and augmentation were applied exclusively to the training folds, whereas validation data remained unchanged to minimize data leakage. All models were initialized from ImageNet-pretrained weights and fine-tuned independently with early stopping (patience = 5). EfficientNet checkpoints were selected according to the highest validation accuracy, whereas ViT and Swin Transformer checkpoints were selected according to the highest validation macro-$F_{1}$ score, consistent with their respective training implementations. Performance metrics, including one-vs-rest multiclass AUC and binary malignant-risk AUC, were reported as mean ± standard deviation across the five folds. Table 10 summarizes the training hyperparameters adopted for each backbone architecture to ensure consistent and reproducible model optimization across all experimental conditions. Prediction-level outputs containing the true and predicted labels for individual validation samples were retained for the RAW, ROS, and DA experiments across the five cross-validation folds. These outputs were used for supplementary prediction-level analyses, including clinically oriented binary performance metrics, confidence interval estimation, OCA-specific sensitivity, and formal fold-wise statistical comparisons. Corresponding prediction-level outputs were not available for the RUS experiments; therefore, RUS was retained in the descriptive comparison but excluded from analyses requiring individual predictions.

Because stratified five-fold cross-validation preserved the original class proportions, the resulting training distributions after each imbalance mitigation strategy were equivalent across folds. Accordingly, Table 11 presents the representative class distribution for a single training partition after applying Random Undersampling (RUS), Random Oversampling (ROS), and Data Augmentation (DA).

Random Undersampling balanced the training data by reducing all classes to the size of the minority class, whereas Random Oversampling replicated minority-class samples until all classes matched the largest class. Data Augmentation increased the effective number of minority-class training samples through online geometric and photometric transformations without modifying the validation data. All balancing procedures were applied exclusively within the training partitions of each cross-validation fold.

### 3.8. Ethical Considerations

The proposed framework is intended exclusively for exploratory research on imbalance-aware oral lesion classification. It should not be interpreted as a clinically validated diagnostic or decision-support system or used as a substitute for professional clinical judgment or definitive diagnosis. All experiments were conducted using de-identified imaging data, and model development followed responsible AI practices, including controlled data partitioning and cross-validation procedures to minimize bias and data leakage. Results should be interpreted as supportive information within a broader clinical assessment rather than as an autonomous diagnostic decision.

### 3.9. Reproducibility and Code Availability

All experiments were implemented in Python 3.10 using PyTorch 2.2.2 for model training and scikit-learn scikit-learn for performance evaluation. Stratified cross-validation was applied to preserve class proportions across folds, and all preprocessing, augmentation, and resampling procedures were conducted exclusively within training partitions to prevent data leakage. Performance metrics were computed consistently across folds and reported as mean ± standard deviation. Random seeds were fixed to ensure deterministic training behavior and reproducible evaluation outcomes. The full codebase, including preprocessing pipelines, training scripts, model configurations, and evaluation utilities, will be made publicly available upon acceptance of this manuscript to facilitate transparency, independent verification, and future research development. All quantitative figures reported in the Section 4 were generated programmatically from the preserved experimental prediction outputs using Python-based analysis and visualization libraries. Confusion matrices, per-class sensitivity plots, and sensitivity-specificity comparisons were computed directly from the corresponding prediction files and numerical results. Figure 1 and Figure 2 are conceptual schematics and contain no experimental measurements. Finally, we promote reproducibility by providing detailed descriptions of the preprocessing pipeline, augmentation protocol, cross-validation strategy, and model training configuration, including dedicated summaries of augmentation parameters and training hyperparameters.

## 4. Results

This section presents the quantitative evaluation of imbalance mitigation strategies across backbone architectures. Multiclass and binary malignant-risk analyses are reported using cross-validation metrics to assess sensitivity, specificity, discrimination capacity, and stability. Results are interpreted with emphasis on clinically meaningful performance differences under imbalanced conditions. Performance comparisons are reported using fold-wise mean and standard deviation and are complemented by formal statistical analyses across matched cross-validation folds. Omnibus comparisons among the available imbalance mitigation strategies were conducted using the Friedman test, followed by paired Wilcoxon signed-rank tests with Holm correction for multiple comparisons and effect-size estimation.

### 4.1. Multiclass Performance Under Imbalance Mitigation Strategies

Table 12 summarizes multiclass classification performance across the evaluated imbalance mitigation strategies (RAW, ROS, RUS, and DA) and model architectures (EfficientNet, Swin Transformer, and Vision Transformer). Performance is reported using accuracy, macro-$F_{1}$ score, AUC, and Cohen’s Kappa, expressed as mean ± standard deviation across cross-validation folds. Overall, the highest metric values were observed for EfficientNet trained with data augmentation (DA), whereas lower performance was generally observed under random under-sampling (RUS). The table provides a comparative overview of model behavior under different class imbalance handling strategies, serving as the basis for the detailed analyses presented in the following sections.

### 4.2. CNN Versus Transformer Architectures

Across imbalance mitigation strategies, convolutional and transformer-based architectures demonstrated distinct performance profiles. Based on the descriptive cross-validation estimates, EfficientNet achieved the highest values across most performance metrics, particularly under augmentation-based training. This suggests that convolutional inductive biases, including localized receptive fields and hierarchical feature aggregation, remain advantageous for oral lesion image classification under limited and imbalanced data conditions.

Transformer-based models, including Swin and ViT, exhibited competitive but slightly lower overall performance. While Swin Transformer approached EfficientNet under DA and ROS strategies, Vision Transformer showed comparatively reduced macro recall and agreement metrics, indicating greater sensitivity to data imbalance and sample variability. Notably, fold-to-fold variability differed across architectures and training strategies, indicating architecture-dependent performance consistency under the evaluated conditions. Overall, EfficientNet showed strong descriptive performance across the evaluated conditions, whereas the transformer models exhibited more architecture-dependent responses to imbalance mitigation.

### 4.3. Resampling Strategies Versus Data Augmentation

Imbalance mitigation strategies exerted a substantial influence on multiclass performance. Random under-sampling (RUS) consistently resulted in the lowest accuracy, macro-$F_{1}$, AUC, and Kappa across architectures, indicating that reducing majority class representation compromised feature learning and class boundary definition. Although RUS produced a balanced training distribution by reducing majority-class samples to the minority-class size, the associated loss of informative majority samples may have contributed to the lower descriptive performance observed across architectures.

Random over-sampling (ROS) provided moderate improvements relative to RAW training, partially enhancing macro recall and agreement metrics. However, gains remained limited, suggesting that simple duplication of minority samples may not sufficiently enrich feature diversity or intra-class variability.

In contrast, DA yielded higher descriptive performance estimates across several evaluated metrics. By synthetically expanding the minority class distribution through geometric and photometric transformations, DA preserved majority class information while increasing effective sample diversity. This condition was associated with higher macro-averaged metrics, AUC values, and Cohen’s kappa estimates, particularly for EfficientNet and Swin Transformer models. Collectively, these findings indicate that augmentation-based strategies provide a more balanced and effective mechanism for mitigating class imbalance in heterogeneous oral lesion classification tasks.

### 4.4. Per-Class Sensitivity Analysis and Error Distribution

**Figure 3:**
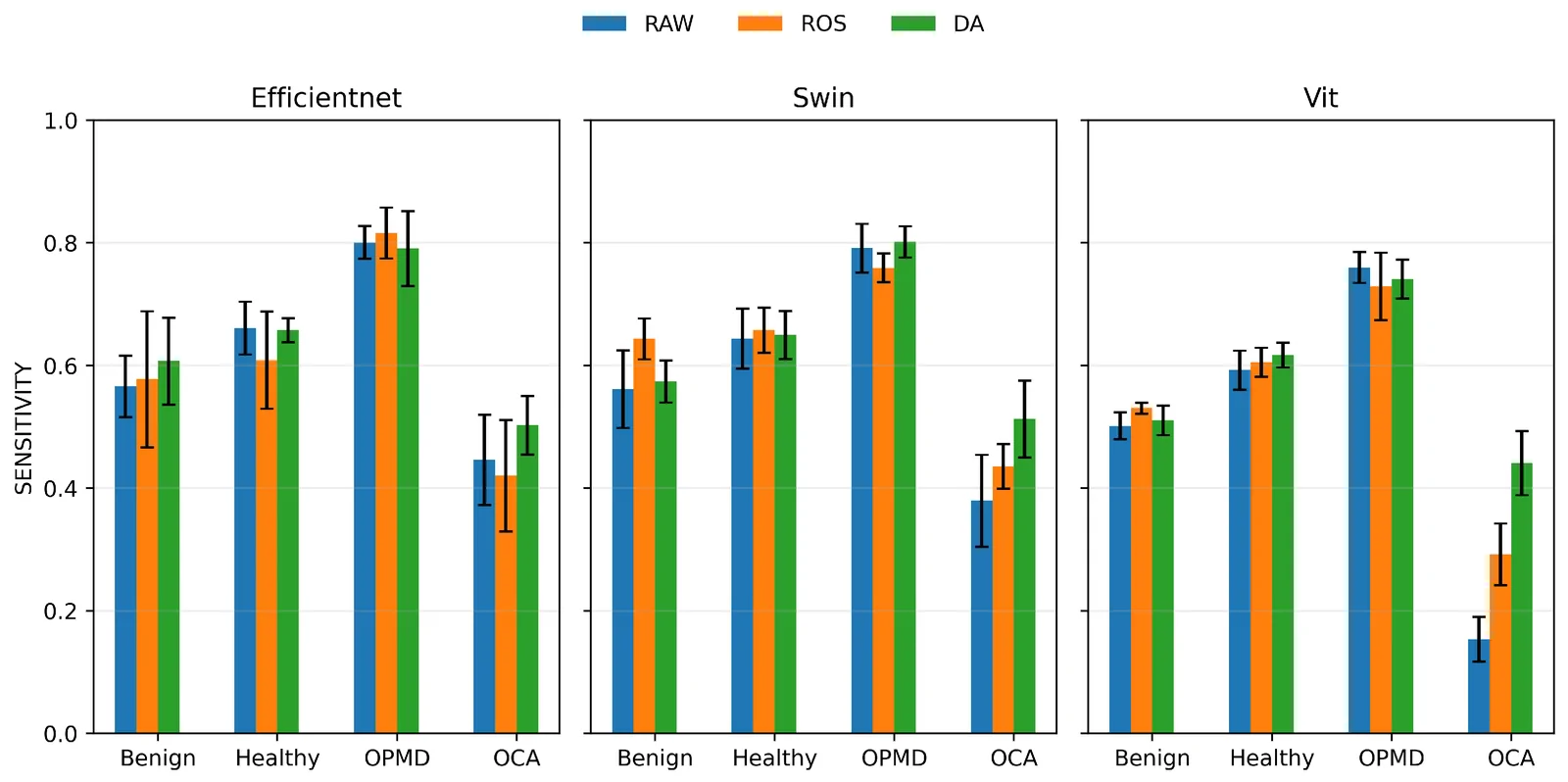
Per-class sensitivity (recall) across imbalance mitigation strategies (RAW, ROS, DA) for EfficientNet, Swin Transformer, and Vision Transformer architectures. Bars represent mean recall across cross-validation folds, and error bars indicate standard deviation. DA generally shows higher OCA, sensitivity across the evaluated architectures while maintaining relatively stable performance in the benign and healthy categories. This pattern suggests improved minority-class representation without substantial degradation of low-risk discrimination. The plotted values and error bars were generated programmatically from the preserved fold-level prediction outputs.

Per-class sensitivity analysis across architectures reveals consistent effects of imbalance mitigation strategies. Data augmentation was associated with higher descriptive OCA sensitivity than RAW and ROS across the three evaluated architectures. In EfficientNet and Swin Transformer, augmentation maintains strong OPMD detection while improving OCA recall, suggesting a descriptive improvement in class discrimination among clinically related lesion categories. The Vision Transformer exhibits the largest relative increase in OCA sensitivity under augmentation, indicating a greater benefit from additional minority-class diversity. Low-risk classes (Benign and Healthy) remain comparatively stable across strategies, with only minor trade-offs observed. In contrast, ROS yields moderate improvements but does not consistently match augmentation performance, particularly for OCA. Overall, augmentation is associated with a more balanced recall distribution across lesion types, supporting its potential utility for multiclass oral lesion classification under imbalanced training conditions. Figure 3 illustrates class-wise sensitivity across imbalance mitigation strategies and backbone architectures.

### 4.5. Binary Clinical Evaluation of Multiclass Models

Although models were trained in a multiclass setting to distinguish among oral lesion categories (healthy, benign, OPMD, and OCA), a clinically oriented binary evaluation was performed to better reflect screening scenarios. In this secondary analysis, oral cancer (OCA) and oral potentially malignant disorders (OPMD) were grouped into a malignant-risk category, while healthy and benign lesions were considered low-risk. This mapping aligns with risk-based triage strategies commonly adopted in early detection settings.

Binary performance was evaluated across cross-validation folds using sensitivity (malignant-risk recall), specificity (low-risk recall), and overall accuracy. Results are reported as mean ± standard deviation (SD) to reflect performance stability. Across architectures, mean sensitivity ranged approximately from 0.74 to 0.82, indicating broadly comparable malignant-risk detection across the evaluated configurations. Specificity values ranged from 0.67 to 0.75, reflecting a moderate trade-off associated with improved malignant-risk detection. Fold-to-fold variability was generally moderate, although its magnitude differed across architectures and training strategies. To provide a more clinically informative assessment of the malignant-risk endpoint, the binary evaluation was extended beyond sensitivity and specificity to include positive predictive value (PPV), negative predictive value (NPV), and $F_{1}$-score. Table 13 summarizes these metrics across the five stratified cross-validation folds. Prediction-level outputs required for this extended analysis were available for RAW, ROS, and DA but not for RUS; therefore, RUS is retained in the descriptive multiclass comparison but excluded from the prediction-level binary analysis.

The extended evaluation demonstrates that the operating characteristics differed across architectures and imbalance mitigation strategies. EfficientNet-ROS achieved the highest mean malignant-risk sensitivity, whereas EfficientNet-DA achieved the highest specificity and $F_{1}$-score among the EfficientNet configurations. Because these values represent descriptive cross-validation estimates, differences among strategies are interpreted together with the formal statistical comparisons reported in the Section 4.8. Across architectures, DA demonstrated a modest but consistent influence on malignant-risk detection compared to raw imbalanced training. In the Swin architecture, sensitivity increased from 0.796 to 0.807, indicating improved identification of high-risk lesions. For ViT, sensitivity remained stable, while EfficientNet showed comparable malignant-risk detection performance between strategies.

Because the malignant-risk endpoint combines OCA and OPMD, its overall sensitivity may obscure performance specifically for the substantially smaller OCA class. To provide a more clinically explicit assessment, OCA was therefore evaluated separately using a one-versus-rest formulation. Table 14 reports the number of correctly detected OCA cases (TP), missed OCA cases (FN), OCA-specific sensitivity, and corresponding 95% Wilson confidence intervals derived from the preserved cross-validation prediction outputs. Prediction-level outputs required for this analysis were available for RAW, ROS, and DA but not for RUS; consequently, RUS was excluded from the OCA-specific prediction-level analysis.

OCA-specific sensitivity was substantially lower than the sensitivity observed for the combined malignant-risk endpoint. Under DA, OCA sensitivity reached 0.503 (95% CI: 0.433–0.572) for EfficientNet, 0.513 (95% CI: 0.443–0.582) for Swin Transformer, and 0.441 (95% CI: 0.373–0.511) for ViT. Nevertheless, DA increased OCA sensitivity relative to RAW for all three architectures, from 0.446 to 0.503 for EfficientNet, from 0.379 to 0.513 for Swin, and from 0.154 to 0.441 for ViT. These results demonstrate that performance for the combined OCA+OPMD malignant-risk endpoint should not be interpreted as equivalent to direct OCA detection and highlight the continuing challenge posed by the limited representation of OCA cases.

Collectively, these findings indicate that DA was associated with higher OCA sensitivity across all three architectures, although the magnitude of improvement varied by architecture and should be interpreted together with the formal statistical comparisons.

**Figure 4:**
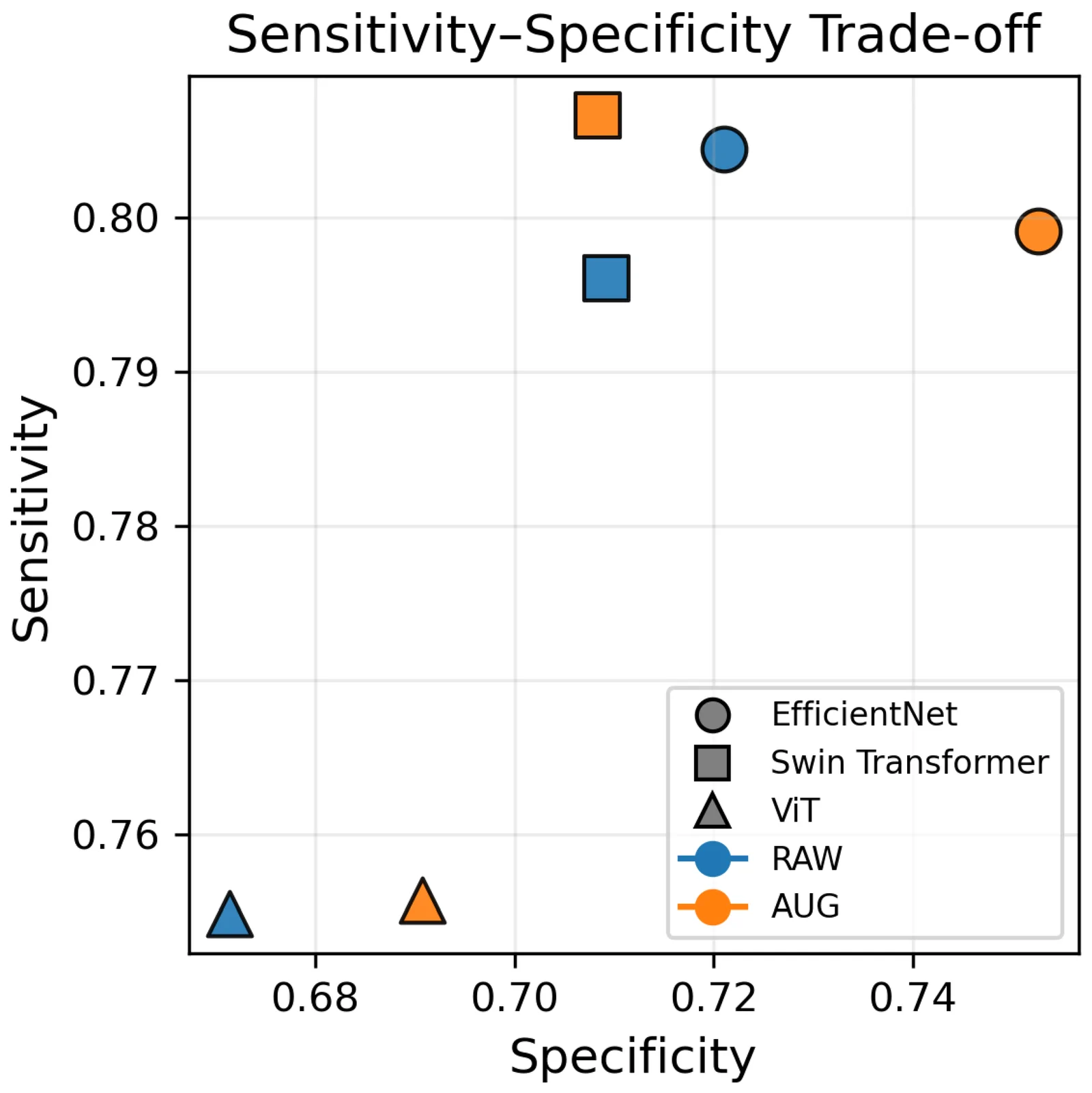
Sensitivity versus specificity trade-off across architectures and training strategies under the binary malignant-risk evaluation setting. Each point represents the mean performance across cross-validation folds. Marker shapes denote architecture (circle: EfficientNet; square: Swin Transformer; triangle: Vision Transformer), while colours indicate training strategy (RAW vs. DA). Models located closer to the upper-right quadrant demonstrate improved screening balance between malignant detection sensitivity and non-malignant specificity. All plotted coordinates were calculated directly from the preserved cross-validation prediction outputs.

To further illustrate the screening trade-off between malignant sensitivity and non-malignant specificity, Figure 4 illustrates the architecture-dependent trade-off between malignant-risk sensitivity and specificity under RAW and DA training.

**Figure 5:**
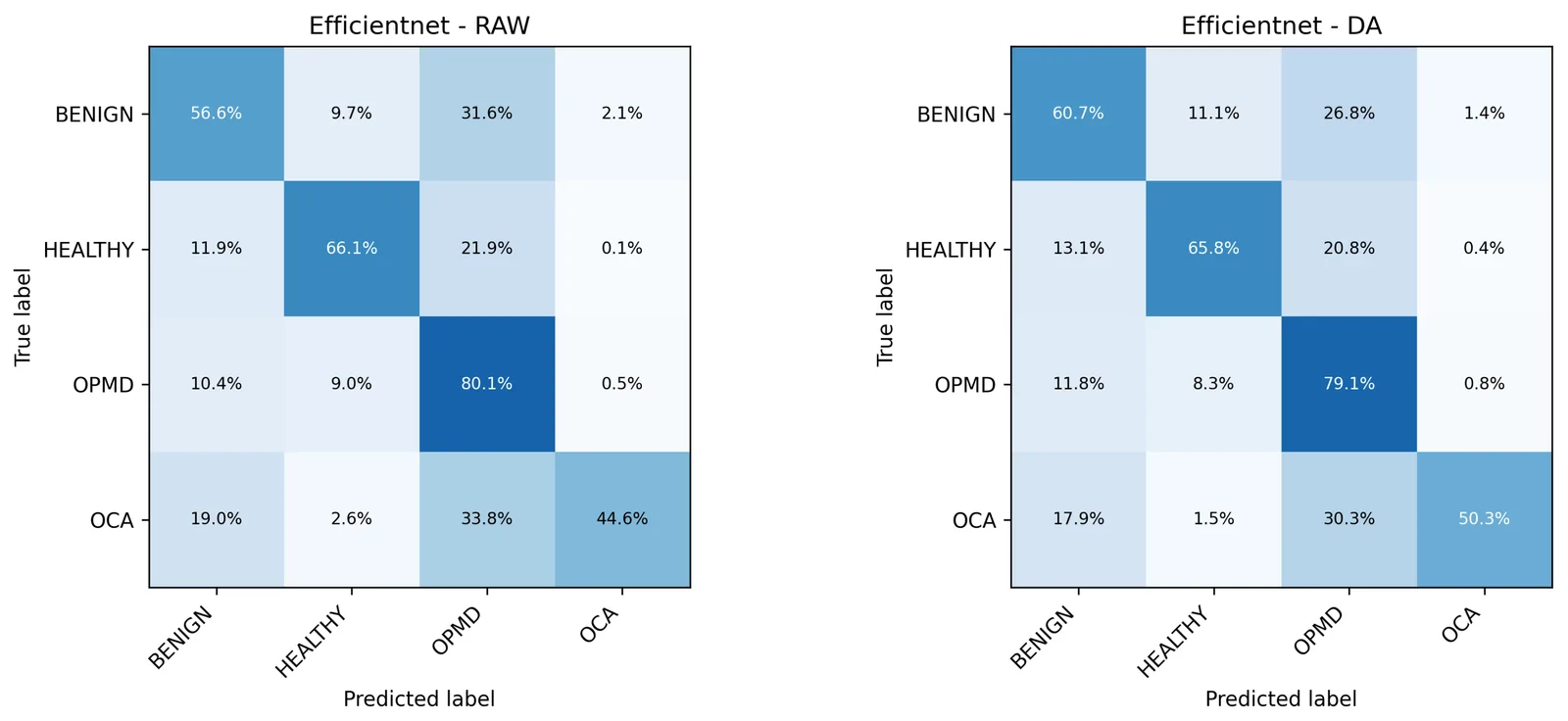
Normalized multiclass confusion matrices for the EfficientNet architecture trained using the RAW and data augmentation (DA) strategies. Values represent row-wise percentages averaged across all cross-validation folds. Data augmentation increased the correct classification of the minority oral cancer (OCA) class from 44.6% to 50.3% while improving benign lesion recognition and maintaining comparable performance for healthy tissue and OPMD. The principal remaining source of error corresponds to confusion between OCA and OPMD, highlighting the intrinsic visual similarity between malignant and potentially malignant oral lesions. The matrices were computed programmatically from the preserved prediction-level outputs.

The multiclass confusion matrices for the best-performing EfficientNet model under the RAW and data augmentation (DA) training strategies are presented in Figure 5. Both models correctly classified the majority of OPMD samples, achieving approximately 80% recall. However, augmentation increased the recognition of the minority OCA class from 44.6% to 50.3%, representing the largest improvement among all diagnostic categories. Simultaneously, the correct classification of benign lesions increased from 56.6% to 60.7%, whereas healthy tissue classification remained largely unchanged. Most residual errors involved confusion between OCA and OPMD, reflecting their visual similarity and partially overlapping clinical characteristics. Overall, the augmented model exhibited a stronger diagonal structure and reduced off-diagonal errors, indicating improved class discrimination while preserving performance for the remaining lesion categories.

### 4.6. Binary Confusion Matrix Analysis

**Figure 6:**
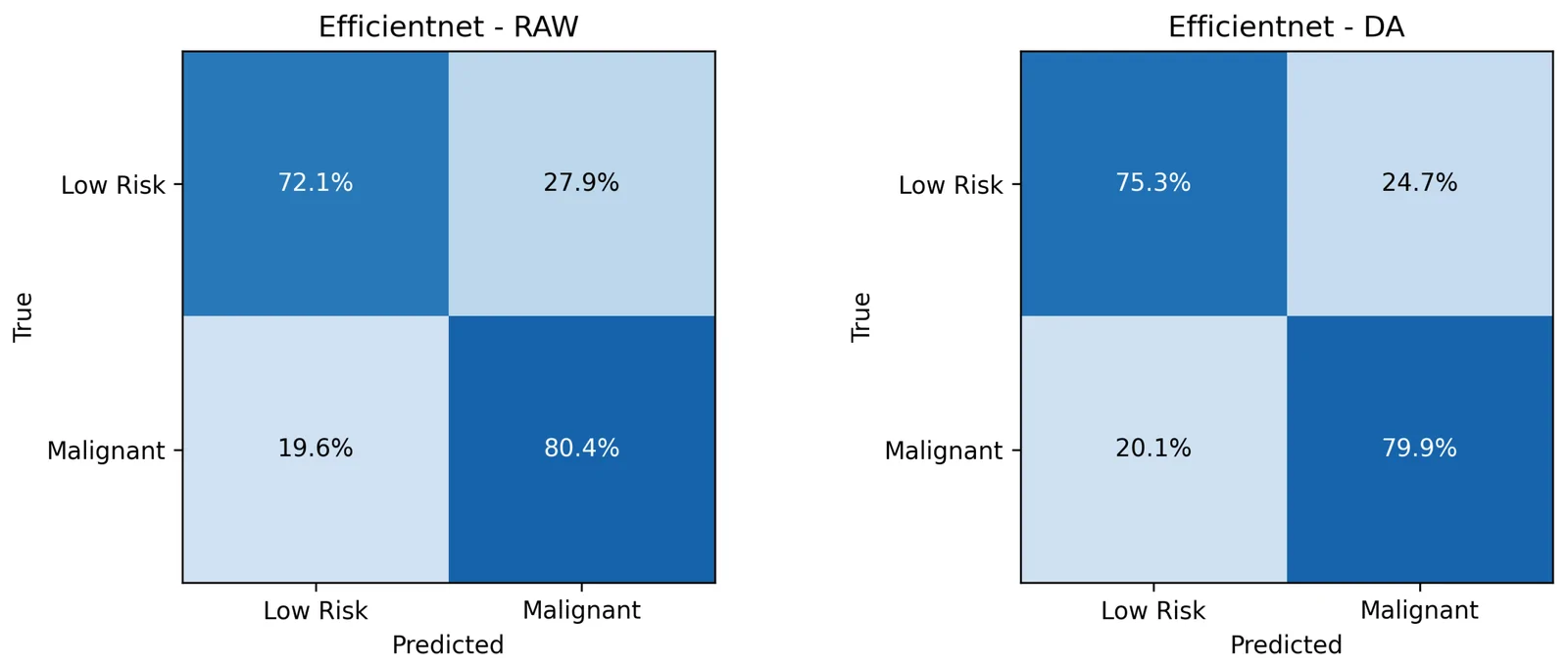
Normalized binary confusion matrices for malignant-risk screening (OCA+OPMD vs. low-risk lesions) using EfficientNet under RAW and DA training. Rows correspond to true class and columns to predicted class. Augmentation maintains high malignant sensitivity while modestly improving low-risk specificity, supporting its relevance for screening-oriented classification. The matrices were computed directly from the preserved prediction-level outputs using the binary OCA+OPMD versus Healthy+Benign mapping.

A clinically oriented binary evaluation was performed by grouping OCA and OPMD as malignant-risk and healthy and benign lesions as low-risk. Sensitivity and specificity were computed across cross-validation folds. Results demonstrated consistent malignant detection with moderate trade-offs in specificity, supporting the screening relevance of augmentation-based training. For EfficientNet, augmentation maintained malignant-risk sensitivity while improving low-risk specificity relative to RAW training. Figure 6 presents the normalized binary confusion matrices for EfficientNet under RAW and augmentation-based training, illustrating screening-oriented performance in terms of malignant-risk detection and low-risk preservation.

### 4.7. Stability and Variability Across Cross-Validation Folds

To examine fold-to-fold consistency under different imbalance mitigation strategies, variability in macro-$F_{1}$ scores was assessed using the reported standard deviations, with lower values indicating more consistent performance across folds. Table 15 presents the mean macro-$F_{1}$ scores and corresponding standard deviations for all architectures and training strategies. DA achieved competitive performance while maintaining relatively low variability, particularly for Vision Transformer and Swin Transformer. By comparison, ROS showed higher variability in several settings, especially for EfficientNet, suggesting greater dependence on fold composition. Descriptively, RUS produced lower mean macro-$F_{1}$ values across all three architectures without a consistent reduction in fold-to-fold variability. Taken together, these findings suggest that augmentation-based training may support both discriminative performance and consistency across folds, a desirable property for imbalanced oral lesion classification.

### 4.8. Statistical Comparison

Formal statistical comparisons were performed across matched cross-validation folds for the RAW, ROS, and DA conditions. Within each architecture, differences among imbalance mitigation strategies were evaluated using the Friedman test, with Kendall’s *W* reported as an effect-size measure. Significant omnibus differences were observed for OCA sensitivity with Swin Transformer (p=0.0408, W=0.64), and for macro-$F_{1}$ (p=0.0408, W=0.64) and OCA sensitivity (p=0.0067, W=1.00) with ViT. The complete omnibus results are reported in Table 16.

Pairwise RAW–DA comparisons were subsequently evaluated using Wilcoxon signed-rank tests with Holm correction for multiple comparisons. None of these pairwise comparisons remained statistically significant after correction. Nevertheless, large rank-biserial effects were observed for macro-$F_{1}$ with EfficientNet ($r_{rb}=0.87$), Swin Transformer ($r_{rb}=0.87$), and ViT ($r_{rb}=1.00$), as well as for OCA sensitivity across all three architectures ($r_{rb}=1.00$). These results indicate directional effects of augmentation, particularly for OCA detection; however, the small number of cross-validation folds limits the statistical power of the pairwise analyses.

### 4.9. Summary of Quantitative Findings

Collectively, the descriptive results showed higher performance estimates under DA for several multiclass and OCA-specific outcomes, while RUS generally produced lower performance and ROS yielded intermediate results. EfficientNet achieved the highest descriptive overall performance, whereas Swin Transformer showed competitive results under augmentation. Formal statistical analysis identified significant omnibus differences for selected architecture–metric combinations, particularly OCA sensitivity; however, RAW–DA pairwise comparisons did not remain statistically significant after Holm correction. Accordingly, the observed advantages of augmentation should be interpreted as directional performance improvements rather than evidence of statistical superiority. The OCA-specific analysis further demonstrated that direct OCA detection remains substantially more challenging than detection of the combined OCA+OPMD malignant-risk category.

## 5. Discussion

### 5.1. Impact of Imbalance Mitigation on Multiclass and Clinical Performance

The results demonstrate that class imbalance mitigation influences both multiclass classification and clinically oriented malignant-risk detection. Overall, data augmentation produced competitive performance across the evaluated architectures and generally improved macro-$F_{1}$ and malignant-risk sensitivity compared with the remaining imbalance mitigation strategies. In contrast, Random Undersampling consistently reduced performance, whereas Random Oversampling yielded more modest improvements. These findings agree with previous studies showing that medically informed data augmentation improves generalization by increasing sample diversity while preserving diagnostically relevant image characteristics [15,28]. Unlike conventional resampling, augmentation introduces realistic image variability that may promote more discriminative feature learning. Although recent oral cancer studies have primarily compared network architectures or overall accuracy, this work systematically evaluates multiple imbalance mitigation strategies under a unified experimental framework. Nevertheless, direct numerical comparisons should be interpreted cautiously because differences in datasets, class distributions, imaging conditions, and validation protocols substantially influence reported performance [16,29]. In relation to recent oral cancer image-classification studies, the present results should be interpreted primarily as complementary rather than directly benchmarked performance. Recent investigations have reported strong classification outcomes using different architectures, datasets, class definitions, and validation designs [34,35,36]. In contrast, the present study emphasizes the comparative effect of imbalance mitigation under fixed cross-validation conditions and extends conventional multiclass evaluation with malignant-risk and OCA-specific analyses. Consequently, numerical differences across studies should not be interpreted as evidence of model superiority because dataset composition, patient-level partitioning, acquisition conditions, and evaluation protocols differ substantially.

### 5.2. Architectural Considerations Under Limited and Imbalanced Data

Architecture choice also influenced robustness under class imbalance. Based on the descriptive cross-validation estimates, EfficientNet showed the strongest overall combination of discriminative performance and fold-to-fold consistency, suggesting that convolutional inductive biases remain advantageous for structured medical image analysis with limited training data. In contrast, transformer-based models, particularly Swin Transformer, benefited substantially from data augmentation but exhibited greater sensitivity to imbalance when trained without mitigation. These findings are consistent with previous studies reporting that CNN-based architectures retain strong performance in small medical imaging datasets because of their parameter efficiency and inductive biases, whereas transformer models generally require larger and more diverse training data to achieve their full potential [19,20]. Consequently, the present results reinforce existing evidence that convolutional architectures remain a suitable choice for small and imbalanced oral imaging datasets, while transformer models derive greater benefit from augmentation strategies that increase effective data diversity.

### 5.3. Clinical Implications and Deployment Considerations

From a clinical perspective, grouping OCA and OPMD into a malignant-risk category reflects screening-oriented triage, where early identification of high-risk lesions is essential. The observed sensitivity–specificity balance suggests that augmentation-based imbalance mitigation may improve malignant-risk detection while maintaining competitive overall performance. These findings agree with recent reviews recommending that AI-assisted oral cancer screening prioritize sensitivity over overall accuracy because delayed detection has important clinical consequences [18,21]. By integrating multiclass and malignant-risk evaluations, the present study provides a clinically oriented assessment of imbalance-aware training. Nevertheless, improved sensitivity may increase false-positive findings, potentially leading to unnecessary referrals, follow-up examinations, biopsies, patient anxiety, and greater healthcare resource utilization. Therefore, the optimal balance between sensitivity and specificity should be established through prospective multicenter studies conducted in representative screening populations before clinical implementation.

### 5.4. Responses to the Research Questions

The experimental findings provide a coherent response to the proposed hypothesis and research questions. Regarding RQ1, data augmentation generally produced competitive performance relative to Random Undersampling and Random Oversampling, particularly in terms of macro-$F_{1}$ and clinically oriented malignant-risk detection. RQ2 showed architecture-dependent changes in malignant-risk sensitivity and specificity under augmentation, with improvements in selected metrics but no uniform advantage across architectures, whereas RQ3 showed architecture-dependent responses, with EfficientNet exhibiting the strongest overall performance and Swin Transformer benefiting from imbalance mitigation. RQ4 further indicated improved recall for the minority OCA class, although performance remained constrained by the limited number of malignant samples. Overall, these findings support the hypothesis that augmentation-based imbalance mitigation can improve clinically relevant classification under small and imbalanced training conditions. Nevertheless, the achieved OCA recall remains insufficient for clinical deployment, and the proposed framework should therefore be regarded as an exploratory methodological study requiring external validation.

### 5.5. Limitations and Future Directions

The principal limitation of this study is that model evaluation was restricted to internal cross-validation using a single publicly available dataset. Because unique patient identifiers were unavailable, partitioning was performed at the image level, and patient-level independence between training and validation folds could not be verified. Thus, although preprocessing, resampling, and augmentation were restricted to the training partitions to prevent procedural leakage, potential leakage arising from multiple images of the same patient across different folds cannot be excluded. Furthermore, the dataset represents a single-country population and smartphone-based acquisition setting. Accordingly, the present results should be interpreted as internal methodological benchmarking rather than evidence of external clinical validity. Independent multicenter datasets with patient identifiers and heterogeneous acquisition conditions are required to evaluate patient-level generalizability and external robustness.

Consequently, the findings should be interpreted within the context of the evaluated dataset, as differences in patient ethnicity, disease prevalence, image acquisition protocols, camera characteristics, illumination conditions, and clinical practice may influence model generalizability. These limitations are consistent with recent reviews identifying single-center datasets, heterogeneous imaging conditions, and limited external validation as persistent challenges in AI-based oral cancer diagnosis [20,21]. External validation using independent multicenter cohorts is therefore essential before clinical deployment.

Second, the present study focused exclusively on representative data-level imbalance mitigation strategies. Algorithm-level approaches, including cost-sensitive learning, focal loss, and domain adaptation, were beyond the scope of this work but represent promising directions for future investigation.

Third, although formal statistical comparisons were added using matched cross-validation folds, the inferential analysis remains limited by the small number of folds and the absence of retained fold-level outputs for the RUS condition. Accordingly, statistical results should be interpreted together with effect-size estimates and descriptive performance. Calibration analysis also remains incomplete, and future validation studies should incorporate reliability diagrams, Expected Calibration Error (ECE), and prospective probability calibration.

Fourth, the possibility of shortcut learning cannot be excluded. Deep learning models may exploit demographic characteristics, anatomical location, image acquisition conditions, or recruitment-related visual cues correlated with diagnostic labels rather than lesion morphology itself. Evaluating robustness across diverse patient populations, imaging protocols, and healthcare settings, together with explainable AI techniques and prospective clinical studies, will be essential to establish the reliability and clinical applicability of imbalance-aware oral cancer classification systems.

Overall, the proposed framework demonstrates promising methodological performance; however, these findings should be interpreted as exploratory rather than evidence of clinical readiness. External multicenter validation, patient-level evaluation, calibration assessment, and prospective clinical studies remain essential before routine clinical implementation.

## 6. Conclusions

### 6.1. Principal Findings

This study systematically evaluated convolutional and transformer-based architectures under multiple imbalance mitigation strategies for multiclass oral lesion classification. The evaluated augmentation strategy generally improved multiclass classification performance for several architectures while maintaining competitive malignant-risk detection. However, these effects were not uniform across all evaluated architectures and performance metrics and should be interpreted as descriptive observations rather than evidence of statistical superiority. Per-class analyses indicated higher sensitivity for malignant-spectrum lesions, particularly OCA, while maintaining stable performance for low-risk categories. Binary malignant-risk evaluation further indicated architecture-dependent sensitivity–specificity trade-offs under augmentation, with improvements in selected clinically relevant metrics but no uniform advantage across architectures.

### 6.2. Clinical and Methodological Implications

The results reinforce the importance of considering class imbalance in medical image classification and indicate that augmentation was associated with improved clinically relevant sensitivity in selected settings while maintaining competitive overall discrimination. Descriptively, augmentation provided a competitive balance between performance and fold-to-fold variability across the evaluated architectures. These findings support the incorporation of augmentation strategies when developing AI models from small and imbalanced oral imaging datasets. From a practical perspective, improved malignant-risk detection may enhance the utility of such systems in screening, triage, and educational applications, particularly in settings where early identification of high-risk lesions is a priority.

### 6.3. Clinical Takeaway

Among the evaluated imbalance mitigation strategies, data augmentation generally showed a favorable descriptive balance between malignant-risk sensitivity and classification stability. These findings suggest that augmentation-based training warrants further evaluation as an imbalance mitigation strategy for oral cancer image-classification models developed from small and imbalanced datasets. In future clinical screening contexts, improved detection of high-risk lesions could support earlier referral decisions; however, the associated false-positive burden must be established through prospective evaluation in representative populations. Although not intended to replace clinical judgment, imbalance-aware AI systems may serve as useful decision-support tools in primary care, community screening programs, and resource-constrained settings.

### 6.4. Future Directions

Future research should prioritize external validation using independent multicenter datasets representing diverse patient populations, imaging protocols, and clinical environments to establish the generalizability of the proposed framework. Additional investigations should incorporate patient-level evaluation, calibration analyses, including reliability diagrams and Expected Calibration Error (ECE), and prospective clinical studies to assess the reliability of predicted probabilities and real-world clinical utility. Furthermore, algorithm-level imbalance mitigation strategies, such as cost-sensitive learning, focal loss, and domain-adaptive pretraining, together with explainable AI techniques, may further improve minority-class discrimination and enhance clinical interpretability. Although the proposed framework demonstrates promising methodological performance, it should be regarded as an exploratory research platform. Consequently, comprehensive external validation remains essential before considering its application in screening, triage, primary care, or other routine clinical settings. These findings should therefore be interpreted within the context of internal image-level cross-validation and should not be considered evidence of clinical generalizability or deployment readiness. Future studies should prioritize independent multicenter validation using patient-wise partitioning, heterogeneous acquisition devices, and diverse populations before considering translation to screening or clinical decision-support settings.

## Figures and Tables

**Table 1:** Methodological comparison of previous studies and the proposed approach.

Study	Images	Split	Validation	Classes	Imbalance Handling
[34]	4161	Image-level	Stratified 5-fold CV + held-out test	4	None reported
[35]	1043	Patient-level	Held-out test split	3	None reported
[36]	1470	Patient-level	Held-out test split	2	Not reported
This Study	3000	Image-level	Stratified 5-fold CV	4	RUS, ROS, DA

**Table 2:** Distribution of diagnostic categories in the oral cavity image dataset.

Diagnostic Category	Number of Images	Percentage (%)
Oral Potentially Malignant Disorder (OPMD)	1394	46.5
Benign Lesions	748	24.9
Healthy	729	24.3
Oral Cancer (OCA)	129	4.3
Total	3000	100.0

**Table 3:** Summary of metadata fields available for each sample in the dataset.

Field	Description
Age	Patient age at the time of diagnosis
Sex	Biological sex (male/female)
Diagnosis	Histopathological outcome
Smoking	Current or past smoking status
Alcohol	Alcohol consumption habits
Betel quid	Betel-quid chewing status

**Table 4:** Descriptive statistics by diagnostic category, including demographic and risk factor distributions.

Category	Count	Age	Male (%)	Smoking (%)	Betel Quid (%)	Alcohol (%)
Benign	748	46.9	58.8	10.0	15.0	4.8
Healthy	729	43.3	45.7	7.4	16.6	4.4
OCA	129	61.8	55.0	10.1	76.7	7.0
OPMD	1394	48.6	63.8	11.9	60.2	11.0

**Table 5:** Summary of backbone architectures evaluated in this study.

Model	Architecture Type	Parameters (M)	Key Characteristics
Vision Transformer (ViT) [39]	Pure Transformer	86	Splits images into non-overlapping patches; processes as tokens via self-attention layers; captures long-range dependencies.
Swin Transformer [40]	Hierarchical Transformer	88	Uses shifted window attention for local context and cross-window interactions; scales linearly with image size; effective for classification and dense vision tasks.
EfficientNet [41]	Convolutional Neural Network (CNN)	5.3	Compound scaling of depth, width, and resolution; designed via neural architecture search; achieves high accuracy with fewer parameters.

**Table 6:** Overview of oversampling strategies applied in this study.

Method	Principle	Advantages	Limitations
ROS [14]	Randomly duplicates existing minority class samples until balance is achieved.	Simple to implement; effective for small imbalance ratios.	Risk of overfitting due to repeated samples; does not introduce new information.
RUS [14]	Randomly removes samples from the majority class until class balance is achieved.	Reduces class dominance and training bias; simple and computationally efficient.	Potential loss of informative majority samples; may increase variance and reduce overall model stability.

**Table 7:** Image data augmentation transformation techniques applied in this study.

Technique	Principle	Advantages	Limitations
Geometric (flip, rotation, crop, resize) [15]	Apply spatial transformations to simulate viewpoint and orientation variability.	Improves invariance to camera angle and positioning; simple and effective.	Excessive transformations may distort clinically relevant features.
Photometric (color jitter, brightness, blur, noise) [15]	Modify pixel intensity distributions to mimic real-world lighting or acquisition conditions.	Enhances robustness to illumination changes and noise.	Risk of altering medically significant texture or color cues.

**Table 8:** Augmentation parameters.

Transformation	Parameter	Probability
Horizontal Flip	p=1.0 (when selected)	33.3%
Rotation	$\pm 15^{\circ }$	33.3%
Resized Crop	224×224 (Scale: 0.8–1.0)	33.3%
Brightness (Color Jitter)	0.8–1.2 (±0.2)	33.3%
Contrast (Color Jitter)	0.8–1.2 (±0.2)	33.3%
Saturation (Color Jitter)	0.8–1.2 (±0.2)	33.3%
Hue (Color Jitter)	−0.02–0.02 (±0.02)	33.3%
Gaussian Blur	Kernel 3×3, σ = 0.1–2.0	33.3%
Sharpness	Factor 2	33.3%

**Table 9:** Description of evaluation metrics used in the study.

Metric	Description
Accuracy	Proportion of correctly classified samples over the total number of samples. Provides a general sense of performance but can be misleading in imbalanced datasets.
Precision	Proportion of true positive predictions among all positive predictions, indicating the model’s reliability when predicting a specific class.
Recall (Sensitivity)	Ability of the model to correctly identify all relevant instances of a given class. Crucial in medical diagnostics to minimise false negatives.
$F_{1}$-score	Harmonic mean of precision and recall, offering a balanced measure that is robust in the presence of imbalanced class distributions.
Area Under the ROC Curve (AUC)	Summarises the model’s ability to distinguish between classes across various threshold settings. Higher values indicate better discriminative power.
Cohen’s Kappa	Measures the agreement between predicted and true labels, accounting for agreement occurring by chance. Particularly informative for imbalanced datasets.

**Table 10:** Training hyperparameters used for each evaluated backbone architecture.

Parameter	EfficientNet	Vision Transformer (ViT)	Swin Transformer
Pretrained Weights	ImageNet	vit-base-patch16-224	swin-base-patch4-window7-224
Optimizer	Adam	AdamW (HuggingFace Trainer default)	AdamW (HuggingFace Trainer default)
Initial Learning Rate	$1\times 10^{-4}$	$1\times 10^{-5}$	$1\times 10^{-5}$
Batch Size	16	32	32
Maximum Epochs	100	50	50
Loss Function	Cross-Entropy Loss	Cross-Entropy Loss	Cross-Entropy Loss
Learning-Rate Scheduler	None	None	None
Early Stopping	Yes	Yes	Yes
Patience	5 epochs	5 evaluation epochs	5 evaluation epochs
Best Model Selection	Highest validation accuracy	Highest validation macro-$F_{1}$	Highest validation macro-$F_{1}$
Cross-Validation	Stratified 5-fold	Stratified 5-fold	Stratified 5-fold

**Table 11:** Representative class distribution within a training partition after applying each imbalance mitigation strategy. Because stratified five-fold cross-validation preserved class proportions, the distributions shown are representative of all training folds.

Class	Original Training	RUS	ROS	DA
Healthy	583	103	583	583
Benign	598	103	598	598
OPMD	1115	103	1115	1115
OCA	103	103	1115	1115

**Table 12:** Multiclass performance results across imbalance mitigation strategies and backbone architectures. Values are reported as mean ± standard deviation across five cross-validation folds. Strategies include RAW (no imbalance mitigation), RUS (random under-sampling), ROS (random over-sampling), and DA. Performance metrics comprise overall accuracy, macro-averaged precision, recall, $F_{1}$-score, area under the receiver operating characteristic curve (AUC), and Cohen’s Kappa coefficient. Higher values indicate improved discriminative performance and inter-class agreement.

Strategy	Model	Accuracy	Precision	Recall	$F_{1}$-Score	AUC	Kappa
RAW	Swin	0.680 ± 0.016	0.647 ± 0.025	0.594 ± 0.024	0.612 ± 0.024	0.866 ± 0.003	0.506 ± 0.024
	ViT	0.629 ± 0.020	0.625 ± 0.050	0.502 ± 0.016	0.522 ± 0.016	0.817 ± 0.013	0.419 ± 0.032
	ENet	0.693 ± 0.011	0.694 ± 0.026	0.619 ± 0.022	0.644 ± 0.018	0.854 ± 0.011	0.525 ± 0.018
RUS	Swin	0.484 ± 0.031	0.447 ± 0.025	0.533 ± 0.030	0.450 ± 0.028	0.755 ± 0.020	0.281 ± 0.037
	ViT	0.432 ± 0.014	0.403 ± 0.008	0.477 ± 0.016	0.395 ± 0.009	0.712 ± 0.017	0.222 ± 0.013
	ENet	0.494 ± 0.017	0.443 ± 0.023	0.499 ± 0.022	0.449 ± 0.022	0.741 ± 0.018	0.268 ± 0.020
ROS	Swin	0.692 ± 0.012	0.694 ± 0.034	0.624 ± 0.010	0.647 ± 0.013	0.871 ± 0.005	0.529 ± 0.018
	ViT	0.631 ± 0.028	0.599 ± 0.052	0.539 ± 0.020	0.558 ± 0.028	0.826 ± 0.010	0.434 ± 0.034
	ENet	0.691 ± 0.022	0.713 ± 0.039	0.601 ± 0.040	0.633 ± 0.040	0.859 ± 0.017	0.517 ± 0.037
DA	Swin	0.696 ± 0.016	0.673 ± 0.028	0.635 ± 0.023	0.650 ± 0.023	0.876 ± 0.005	0.531 ± 0.022
	ViT	0.641 ± 0.017	0.598 ± 0.011	0.578 ± 0.007	0.586 ± 0.007	0.836 ± 0.006	0.450 ± 0.023
	ENet	0.706 ± 0.022	0.699 ± 0.023	0.652 ± 0.029	0.669 ± 0.024	0.881 ± 0.015	0.545 ± 0.037

**Table 13:** Binary malignant-risk classification performance across five stratified cross-validation folds. OCA and OPMD were grouped as positive cases, whereas Healthy and Benign were grouped as negative cases. Values are reported as mean ± standard deviation across folds.

Model	Strategy	Sensitivity	Specificity	PPV	NPV	$F_{1}$
EfficientNet	RAW	0.804 ± 0.021	0.721 ± 0.037	0.749 ± 0.049	0.784 ± 0.035	0.774 ± 0.017
EfficientNet	ROS	0.817 ± 0.030	0.719 ± 0.044	0.752 ± 0.051	0.797 ± 0.038	0.781 ± 0.019
EfficientNet	DA	0.799 ± 0.061	0.753 ± 0.067	0.773 ± 0.069	0.789 ± 0.062	0.783 ± 0.014
Swin	RAW	0.796 ± 0.039	0.709 ± 0.043	0.740 ± 0.037	0.774 ± 0.036	0.766 ± 0.024
Swin	ROS	0.760 ± 0.050	0.761 ± 0.036	0.769 ± 0.038	0.758 ± 0.044	0.762 ± 0.027
Swin	DA	0.807 ± 0.027	0.708 ± 0.015	0.741 ± 0.014	0.781 ± 0.026	0.772 ± 0.016
ViT	RAW	0.755 ± 0.020	0.671 ± 0.034	0.704 ± 0.030	0.728 ± 0.025	0.728 ± 0.014
ViT	ROS	0.736 ± 0.028	0.710 ± 0.048	0.725 ± 0.043	0.725 ± 0.035	0.729 ± 0.019
ViT	DA	0.756 ± 0.031	0.691 ± 0.031	0.717 ± 0.025	0.734 ± 0.030	0.735 ± 0.015

**Table 14:** OCA-specific detection performance based on preserved cross-validation prediction outputs. OCA was evaluated using a one-versus-rest formulation. Sensitivity is reported with a 95% Wilson confidence interval.

Model	Strategy	TP	FN	OCA Sensitivity	95% CI
EfficientNet	RAW	87	108	0.446	[0.378–0.516]
EfficientNet	ROS	82	113	0.421	[0.353–0.491]
EfficientNet	DA	98	97	0.503	[0.433–0.572]
Swin	RAW	74	121	0.379	[0.314–0.449]
Swin	ROS	85	110	0.436	[0.368–0.506]
Swin	DA	100	95	0.513	[0.443–0.582]
ViT	RAW	30	165	0.154	[0.110–0.211]
ViT	ROS	57	138	0.292	[0.233–0.360]
ViT	DA	86	109	0.441	[0.373–0.511]

**Table 15:** Cross-validation stability analysis based on macro-$F_{1}$ score variability. Lower standard deviation indicates greater fold-to-fold consistency.

Strategy	Model	Macro-$F_{1}$ Mean	SD
RAW	Swin	0.612	0.024
RAW	ViT	0.522	0.016
RAW	ENet	0.644	0.018
RUS	Swin	0.450	0.028
RUS	ViT	0.395	0.009
RUS	ENet	0.449	0.022
ROS	Swin	0.647	0.013
ROS	ViT	0.558	0.028
ROS	ENet	0.633	0.040
DA	Swin	0.650	0.023
DA	ViT	0.586	0.007
DA	ENet	0.669	0.024

**Table 16:** Friedman omnibus comparisons of selected performance metrics across the available imbalance mitigation strategies (RAW, ROS, and DA) using matched cross-validation folds. Kendall’s *W* is reported as an effect-size measure.

Architecture	Metric	Friedman $\chi^{2}$	*p*-Value	Kendall’s *W*
EfficientNet	Macro-$F_{1}$	5.20	0.0743	0.52
EfficientNet	OCA Sensitivity	3.60	0.1653	0.36
Swin	Macro-$F_{1}$	3.60	0.1653	0.36
Swin	OCA Sensitivity	6.40	0.0408	0.64
ViT	Macro-$F_{1}$	6.40	0.0408	0.64
ViT	OCA Sensitivity	10.00	0.0067	1.00

## Data Availability

The dataset used in this study is publicly archived in the Zenodo repository as Dataset of Annotated Oral Cavity Images for Oral Cancer Detection (DOI: 10.5281/zenodo.10664056; all versions DOI: 10.5281/zenodo.10664055). The dataset is distributed under the Creative Commons Attribution–NonCommercial–NoDerivatives 4.0 International (CC BY-NC-ND 4.0) license. The repository record is publicly accessible; however, access to the image files is restricted. Researchers may request access through Zenodo using an institutional account by providing a brief description of the intended research, the principal investigator’s affiliation, and agreeing to the dataset’s non-commercial use conditions and citation requirements established by the dataset authors.

## Abbreviations

The following abbreviations are used in this manuscript:

AI	Artificial Intelligence
DA	Data Augmentation
OCA	Oral Cancer
OPMD	Oral Potentially Malignant Disorder
ViT	Vision Transformer
